# The Physician’s Visiting List, Diary, and Book of Engagements for 1853

**Published:** 1852-11

**Authors:** 


*The Physician's Visiting List, Diary, and Book of Engagements for* 1853. *Philadelphia,* LINDSAY & BLAKISTON.

We have received from the publishers, the second annual with the above title. In the preface, they say:

The publishers were first induced to prepare this little work, from the frequent inquiries they had for something of the kind, and at the recommendation of numerous physicians: its very favorable reception by the profession has afforded them much gratification and encouragement. With it in his pocket, the practitioner has always by him a list of patients, his professional engagements, and his day-book, as well as a diary, or memoranda.—They intend continuing it from year to year, and they trust it may be found so indispensable, that after once using it, physicians will never again be without it. Copies can be had, prepared for *twenty-five* or *fifty* patients per week.

No physician who has once used it, would willingly be without it. J.

